# Progressive steps and catalytic cycles in methanol-to-hydrocarbons reaction over acidic zeolites

**DOI:** 10.1016/j.fmre.2021.08.002

**Published:** 2021-08-16

**Authors:** Liu Yang, Chang Wang, Weili Dai, Guangjun Wu, Naijia Guan, Landong Li

**Affiliations:** a School of Materials Science and Engineering, Nankai University, Tianjin 300350, China; b Frontiers Science Center for New Organic Matter and Key Laboratory of Advanced Energy Materials Chemistry of Ministry of Education, College of Chemistry, Nankai University, Tianjin 300071, China

**Keywords:** Methanol-to-hydrocarbons, H-ZSM-5 zeolite, Reaction mechanism, First carbon-carbon bond, Aldol-cycle

## Abstract

Understanding the complete reaction network and mechanism of methanol-to-hydrocarbons remains a key challenge in the field of zeolite catalysis and C1 chemistry. Inspired by the identification of the reactive surface methoxy species on solid acids, several direct mechanisms associated with the formation of the first C-C bond in methanol conversion have been recently disclosed. Identifying the stepwise involvement of the initial intermediates containing the first C-C bond in the whole reaction process of methanol-to-hydrocarbons conversion becomes possible and attractive for the further development of this important reaction. Herein, several initial unsaturated aldehydes/ketones containing the C-C bond are identified *via* complementary spectroscopic techniques. With the combination of kinetic and spectroscopic analyses, a complete roadmap of the zeolite-catalyzed methanol-to-hydrocarbons conversion from the initial C-C bonds to the hydrocarbon pool species *via* the oxygen-containing unsaturated intermediates is clearly illustrated. With the participation of both Brønsted and Lewis acid sites in H-ZSM-5 zeolite, an initial aldol-cycle is proposed, which can be closely connected to the well-known dual-cycle mechanism in the methanol-to-hydrocarbons conversion.

## 1 Introduction

As a sustainable route to obtain basic chemicals, methanol-to-hydrocarbons (MTH) conversion over acidic zeolites has attracted extensive attention [[1], [2], [3], [4], [5]]. The reaction network of MTH is very complicated and understanding the mechanism of MTH reaction is always a challenging topic in C1 chemistry. Recently, the direct reaction mechanism involving the formation of the first C-C bond in the initial period of MTH has again triggered significant research interest [[6], [7], [8], [9], [10], [11], [12]].

Inspired by the recent work of Copéret [8], Lercher [9], and their colleagues, where a Koch-type carbonylation mechanism was proposed, the acetate species associated with the formation of first C-C bond were postulated. Further spectroscopic and theoretical investigations on the formation of acetate species and the process of methanol carbonylation were performed thereafter [10,13]. Our recent work indicated that acetaldehyde formed from the hydrogenation of surface-bound acetyl species was responsible for initiating the MTH conversion on H-ZSM-5 zeolite [14]. Several other direct mechanisms based on the reactive surface methoxy species (SMS) were proposed in parallel [11,12]. Subsequently, the transformation from the direct to the indirect mechanism, i.e., hydrocarbon pool (HCP) or dual-cycle mechanism, was investigated *via* spectroscopic and theoretical strategies [13,15,16].

Referring back to the Koch-type carbonylation route, CO is the key intermediate, which can be formed from methanol dehydrogenation *via* formaldehyde intermediate in the presence of Al-OH or extra-framework Al (EFAL) species [13,17]. In acidic zeolites, the framework Al species act as Brønsted acid sites (BAS), and the extra-framework Al or the metal cations can act as Lewis acid sites (LAS) [18,19]. For zeolites with the co-existence of BAS and LAS, CO can be formed from methanol dehydrogenation at LAS. Subsequently, acetaldehyde and acetate species, for example acetic acid and methyl acetate, can be formed at BAS *via* the Koch-type carbonylation route [14]. The formed acetaldehyde can induce the formation of HCP species *via* progressive aldol-condensation, hydrogen-transfer and cyclization routes, and subsequently, trigger the MTH conversion. Acetic acid and methyl acetate can also be converted to hydrocarbons at high reaction temperatures like 673 K [9,15], while the stepwise of acetic acid or methyl acetate conversion over acidic zeolites is still not clear. It has been recently revealed that acetic acid can be easily converted to acetone *via* the ketonization reaction even at the weak acidic Si-OH groups [20]. In the presence of LAS, the formed acetone will be further converted to alkenes *via* aldol condensation. Therefore, the participation or even synergy between BAS and LAS is expected to promote the formation of the first C-C bond and the subsequent MTH conversion.

With the co-existence of BAS and LAS, an obvious enhancement of propene selectivity and catalyst lifetime could be achieved in the MTH conversion [21,22]. This might be due to the reduction of Brønsted acid site density, thus suppressing the aromatic cycle. Very recently, Van Speybroeck and colleagues proposed a supramolecular view of MTH conversion on the cooperative role of BAS and LAS in alkaline-earth metal modified H-ZSM-5 zeolites following the previous work of Bailleul et al. [19]. Meanwhile, Zheng and colleagues demonstrated that the synergistic effect between BAS and LAS could promote the initial C-C bond formation in the MTH conversion *via* DFT calculations [18]. However, a clear roadmap of zeolite-catalyzed MTH conversion from the initial C-C bonds to the HCP species *via* the oxygen-containing unsaturated intermediates with the involvement of both BAS and LAS is still not known.

In this study, the first C-C bond containing intermediates, namely acetaldehyde, acetic acid and methyl acetate, were identified over H-ZSM-5 zeolite. Acetone, as an active intermediate of acetic acid and acetaldehyde, was also confirmed. According to kinetic and spectroscopic analyses, the roles of the oxygen-containing unsaturated intermediates in the MTH conversion were established, deriving a clear roadmap of MTH conversion catalyzed by H-ZSM-5 zeolite with the participation of both BAS and LAS.

## 2 Experimental section

### 2.1 Materials and reagents

The H-ZSM-5 zeolite with an *n*SiO_2_/*n*Al_2_O_3_ ratio of 28 was provided by Sinopec Co. Methanol (99.8 %, CAS: 67-56-1) was purchased from Acros, acetaldehyde (99%, CAS: 75-07-0), acetic acid (99.5%, CAS: 64-19-7) and acetone (99.5%, CAS: 67-64-1) were from Aladdin. ^13^C-methanol (99 % ^13^C, CAS: 1472-26-8) was from Cambridge Isotope Laboratories and acetone-2-^13^C (99 % ^13^C, CAS: 3881-06-9) was from Sigma-Aldrich.

### 2.2 Reaction of MTH conversion

The isothermal MTH conversion was performed in a fixed-bed reactor at atmospheric pressure. Typically, 0.4 g H-ZSM-5 sample (sieve fraction, 0.25–0.5 mm) was placed in a quartz reactor (5 mm i.d.) and activated under flowing helium at 723 K for 1 h. After cooling to the desired reaction temperature, methanol was pumped into the catalyst bed with a weight hourly space velocity (WHSV) of 1.0 /h. The products were analyzed by an on-line gas chromatograph Shimadzu GC-2010 plus with a flame ionization detector (FID) and a Poraplot Q-HT column (40 m × 0.18 mm × 0.18 μm) to separate hydrocarbons.

The temperature-programmed surface reaction (TPSR) of methanol conversion (without and with cofeeding reagent) was also performed in the aforementioned fixed-bed reactor connected with a downstream gas sampling mass spectrometer (MS, Pfeiffer Omnistar). Typically, helium was utilized as the carrier gas because of its low m/z value of 4, which has no overlap with the main products or intermediates in the reaction. The gas phase products were on-line analyzed by MS referring to the database of the National Institute of Standards and Technology (NIST). In the case of ^13^C-enriched methanol conversion, the m/z values of the reactant and some products were referred to as follows: methanol (32), hydrogen (2), helium (4), carbon monoxide (13), ethylene (29), propylene (42), acetaldehyde (46), dimethyl ether (47), acetic acid (62), acetone (61), 2-methyl-2-cyclopenten-1-one (72), and methyl acetate (77). In the case of ^12^C-acetic acid conversion, the m/z values of the reactant and some products were referred to as follows: acetic acid (60), acetone (58), CO_2_ (44), and water (18). Additionally, all of the above data were obtained by referring to the value of helium in order to eliminate the error caused by unstable carrier gas. More details are available in the Supporting Information (Table S1) [23].

### 2.3 *In situ* UV-vis spectroscopy

The *in situ* UV-vis spectra were recorded in the diffuse reflection mode in the range of 200-600 nm using an AvaSpec-2048 fiber optic spectrometer, an AvaLight-DH-S deuterium light source by Avantes, and a glass fiber reflection probe HPSUV1000A by Oxford Electronics. Before starting the MTH conversion, the glass fiber reflection probe was placed in the fixed-bed reactor on the top of the catalyst with a gap of ∼1.0 mm [24]. Reference UV-vis spectra of the fresh catalysts were recorded at the reaction temperature of 523 K prior to starting the methanol flow.

### 2.4 ^13^C CP MAS NMR and GC-MS analyses

The nature of occluded organic species in spent catalysts after MTH conversion was analyzed by GC-MS and ^13^C CP MAS NMR spectroscopy. ^13^C CP MAS NMR measurements were performed on a Bruker Avance III 400WB spectrometer at the resonance frequency of 100.6 MHz, with the contact time of 3 ms, the repetition time of 4 s, and the sample spinning rate of 8.0 kHz. To avoid contact with air, all spent catalyst samples were transferred from the reactor into the gas-tight MAS NMR rotors inside a glove box purged with dry nitrogen. The ^13^C chemical shifts are referenced to adamantane (38.5 ppm). The details of GC-MS analyses are available in our previous work [14]. Typically, 0.1 g of spent catalyst sample was carefully dissolved in 1 M HF solution. This solution was treated with CH_2_Cl_2_ to extract the organic compounds and the residual water was removed by adding sufficient sodium sulfate solid. Then, 0.2 μL of the organic extract was analyzed by GC-MS (GC-MS-QP2010 SE) with a RXI-5MS column (30 m, 0.25 mm i.d., stationary phase thickness 0.25 μm). The following temperature program was employed, i.e., isothermal heating at 313 K for 6 min, heating to 553 K with a rate of 10 K/min, and isothermal heating at 553 K for 10 min.

## 3 Results and discussion

### 3.1 Oxygen containing unsaturated species in the initial period of MTH conversion

**Fig. 1:**
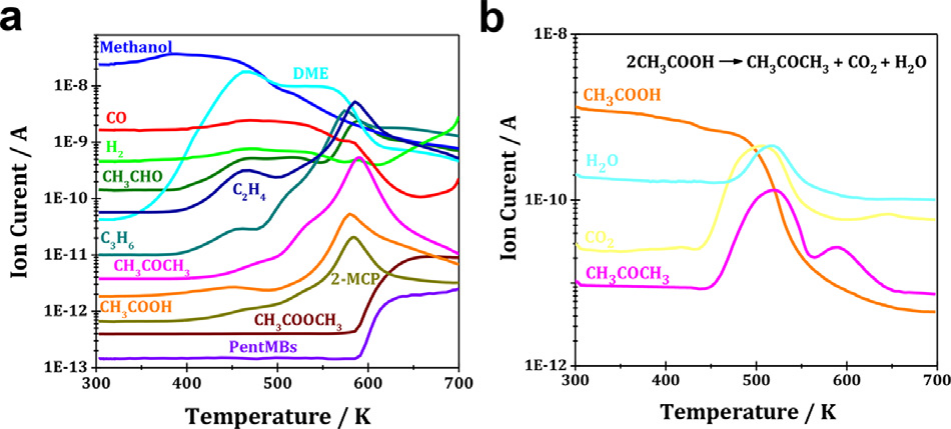
Temperature-programmed surface reaction profiles of (a) ^13^C-enriched methanol and (b) acetic acid (CH_3_COOH) conversion with a constant flow over H-ZSM-5 catalyst. Reaction conditions: WHSV = 1.0 /h.

H-ZSM-5 zeolite with the co-existence of BAS and LAS was utilized as the model catalyst for MTH conversion (see Fig. S1 and corresponding discussion in Supporting Information for details). According to previous reports [9,10,14], the possible intermediates containing C-C bond, namely acetic acid, methyl acetate and acetaldehyde, formed in the initial period of MTH conversion *via* methanol carbonylation, were identified by methanol TPSR. As shown in Fig. 1a, CO and H_2_ were detected as the main products in the very early stage of MTH conversion, revealing the occurrence of methanol dehydrogenation, in line with the previous studies [9,13,14,17,25]. With the progress of reaction, DME appeared as the dominate product and subsequently reached equilibrium with methanol. With the further progress of reaction, acetaldehyde and acetic acid started to appear. Acetaldehyde from the hydrogenation of surface-bound acetyl species was responsible for triggering the MTH conversion on H-ZSM-5 zeolite [14]. Therefore, higher amounts of ethene and propene with a quite similar variation trend as acetaldehyde were simultaneously detected. Additionally, a notable amount of methyl acetate started to appear when the reaction temperature was increased to >583 K, much later than those of ethene and propene. That is, methyl acetate was not responsible for the formation of lower olefins at low reaction temperatures. However, at higher reaction temperatures of >573 K, methyl acetate could be converted into aromatics and accordingly facilitate the MTH reaction [14,15]. Moreover, 2-methyl-2-cyclopenten-1-one (2-MCP) from acetaldehyde condensation and cyclization appeared simultaneously [14,26]. With further increasing reaction temperature to over 583 K, the 2-MCP curve started to slope down, while simultaneously, pentamethylbenzenes (pentaMBs) started to appear and the curve slopped up instead. According to the previous reports [14,27,28], 2-MCP was an important intermediate to generate aromatics, thus triggering the aromatic cycle for MTH reaction.

Interestingly, a significant amount of acetone could also be observed. Our previous observations demonstrated that the ketonization of acetic acid to acetone could easily take place even on very weak acidic Si-OH groups [20]. Therefore, acetone detected herein should originate from acetic acid ketonization at BAS of H-ZSM-5 zeolite, which could be well supported by TPSR profiles of acetic acid conversion. As shown in Fig. 1b, a slight slope down of the acetic acid curve was observed at low temperatures, probably due to its adsorption and interaction with H-ZSM-5 zeolite. With increasing reaction temperature to over 453 K, a steep slope down of the acetic acid curve occurred, and simultaneously, a large amount of acetone, CO_2_ and H_2_O started to appear, indicating acetic acid ketonization to acetone over H-ZSM-5 zeolite. Therefore, the roles of acetic acid in the MTH conversion at the reaction temperature of > 523 K could be reflected by the behaviors of acetone. In addition, the formed acetaldehyde could be converted to acetone *via* the aldol-condensation pathway [29,30], which could be well supported by on-line MS monitoring of acetaldehyde conversion over H-ZSM-5 under study. As shown in Fig. S2, acetone, CO_2_ and H_2_ were observed in the initial period of acetaldehyde conversion. Considering the high reactivity of acetaldehyde, acetic acid and acetone over the acidic zeolites, their roles in the MTH conversion require further identification.

### 3.2 Roles of the oxygen containing unsaturated species in the MTH conversion

**Fig. 2:**
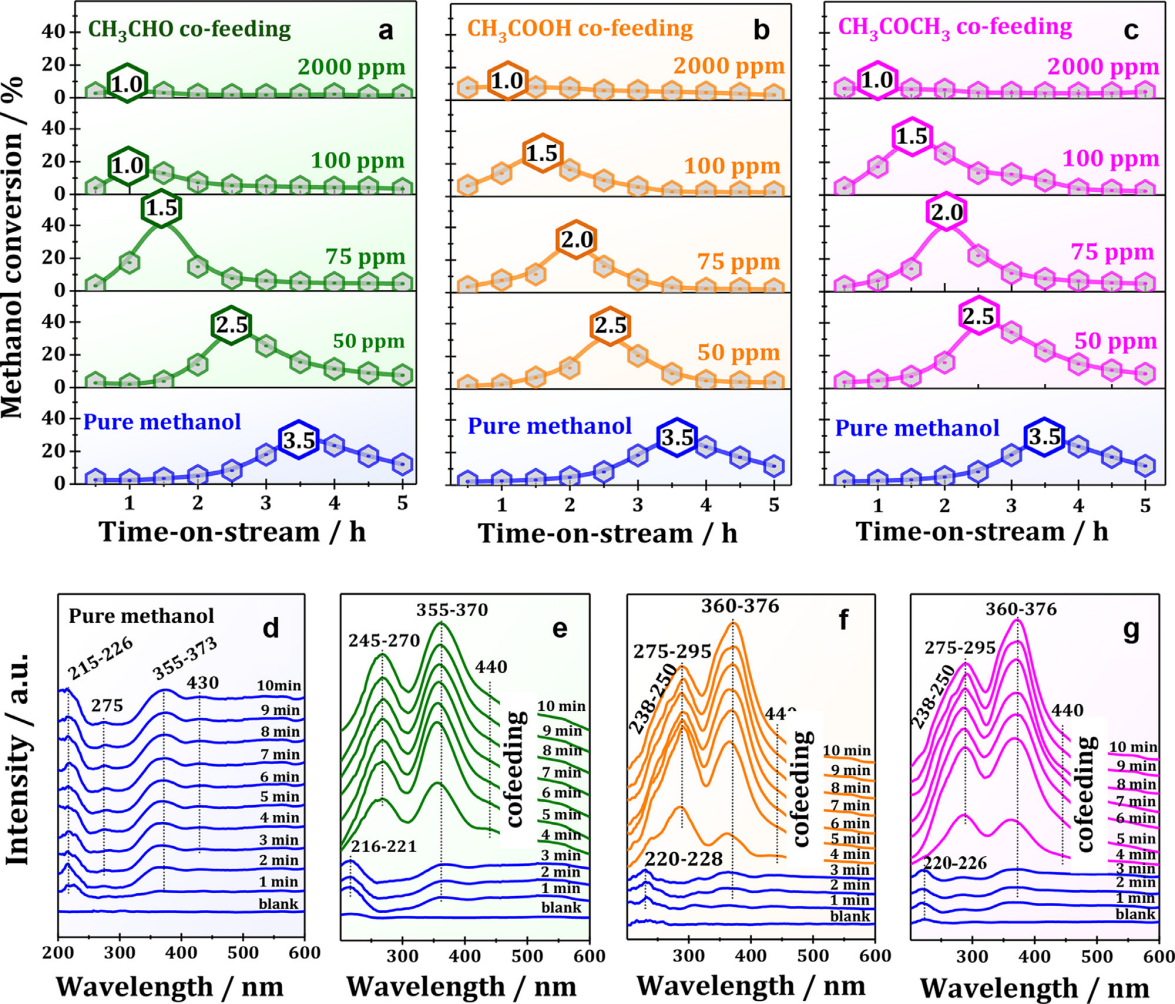
Methanol conversion over H-ZSM-5 zeolite at 523 K and TOS = 5 h (a) without (blue) and with (green) acetaldehyde, (b) (orange) acetic acid or (c) (magenta) acetone co-feeding. *In situ* UV-vis spectra recorded during the MTH conversion (d) without and (e) with (green) acetaldehyde, (f) (orange) acetic acid or (g) (magenta) acetone co-feeding at TOS = 10 min (For interpretation of the references to color in this figure legend, the reader is referred to the web version of this article.).

Co-feeding experiments were first performed to clarify the roles of the aforementioned oxygen containing unsaturated species, namely acetaldehyde, acetic acid and acetone, in the early stages of MTH conversion at low reaction temperature of 523 K. As shown in Fig. 2a–c, with acetaldehyde, acetic acid or acetone co-feeding (50–2000 ppm), the induction period of the MTH conversion could be significantly shortened from 3.5 to 1.0 h. However, with increasing the amounts of co-feeding species up to 2000 ppm, a sharp decline in methanol conversion could be observed. This is due to the strong chemisorption of the aforementioned species, thus making the BAS inaccessible to the methanol molecules. Additionally, a quite similar variation trend of methanol conversion could be observed after acetic acid or acetone co-feeding, hinting that acetic acid or acetone co-feeding could produce similar effects on the induction period of the MTH conversion. This is in line with the acetic acid TPSR profiles (Fig. 1b), i.e., acetic acid could be rapidly converted to acetone at 523 K. Therefore, the effects of acetic acid on the MTH conversion should be roughly equivalent to those of acetone.

For more information on the roles of the aforementioned oxygen containing unsaturated species in the MTH conversion, *in situ* UV-vis experiments were performed during MTH conversion without and with acetaldehyde, acetic acid or acetone co-feeding, at 523 K and a TOS of 10 min. As shown in Fig. 2d, a dominant band at 215-226 nm assigned to unsaturated aldehydes/ketones (π→π*) occurred in the initial period of MTH conversion. With the progress of MTH conversion, new bands at about 275 and 355-373 nm due to neutral methylated benzenes or unsaturated aldehydes/ketones (n→π*), and unsaturated aldehydes/ketones (n→π*) or methylbenzenium ions, respectively, started to appear [14,31]. With the further increase of TOS to 3 min, additional band at about 430 nm, attributed to trienylic/highly methylated arenium ions, occurred. These results clearly demonstrated that the oxygen containing unsaturated species formed during the initial stage of MTH conversion were rapidly involved in the subsequent aldol condensations and gradually transferred to aromatics, thus promoting the MTH conversion *via* the aromatic cycle, in good agreement with the previous studies [14]. With acetaldehyde, acetic acid or acetone co-feeding, aromatics (bands at 270 and 370 nm) were rapidly formed and became the dominating species, revealing that the induction period of the MTH conversion was significantly shortened, in good consistency with the catalytic results shown in Fig. 2a–c. In addition, similar organic species with the quite similar variation trend could be observed for acetic acid or acetone conversion at 523 K (Fig. S3), revealing that acetic acid or acetone co-feeding could produce similar effects on the period induction of the MTH conversion, also in line with the catalytic results (Fig. 1).

**Fig. 3:**
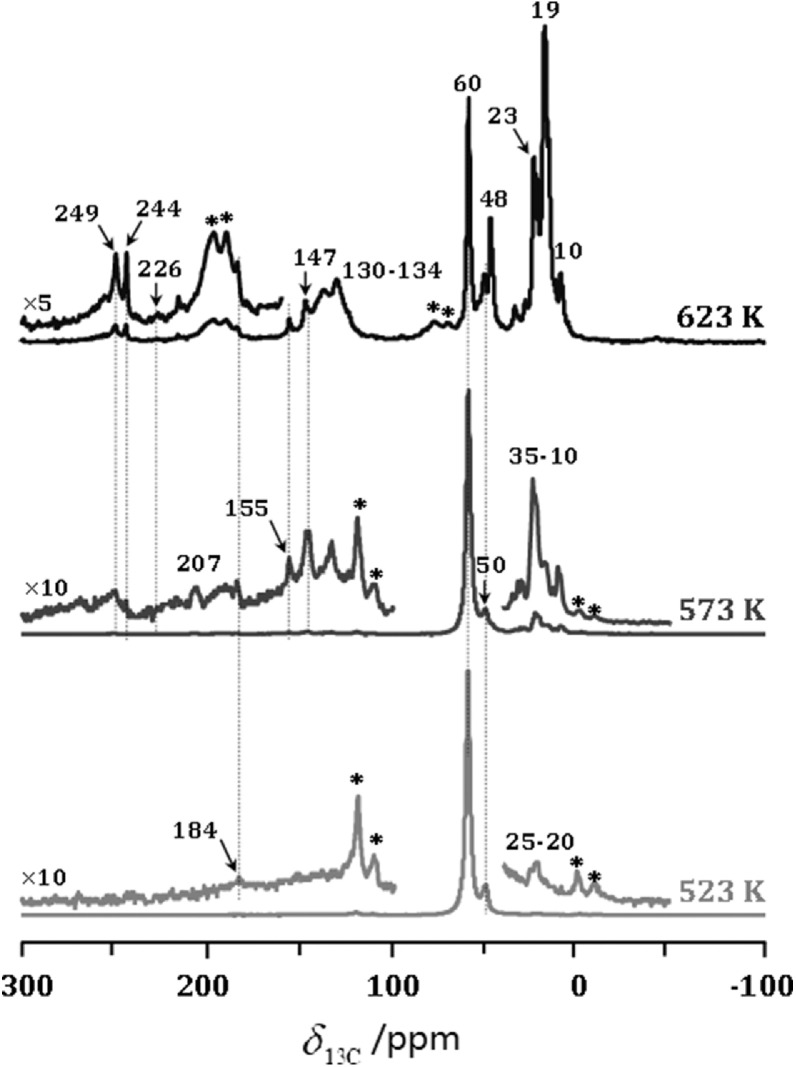
**^13^C MAS NMR spectra of spent H-ZSM-5 catalyst obtained after MTH conversion at TOS of 5 min**. *: spinning sidebands.

To confirm the nature of the organic species formed during the initial process of the MTH conversion without and with acetaldehyde co-feeding over H-ZSM-5 catalyst, ^13^C CP MAS NMR measurements were performed. Fig. 3 shows the ^13^C CP MAS NMR spectra of the spent H-ZSM-5 catalysts after MTH conversion at different temperatures at TOS of 5 min. The adsorbed DME (*δ*_13C_ at 60 ppm) and methanol (*δ*_13C_ at 50 ppm) [32] were observed as the dominant species occluded in H-ZMS-5 catalyst for MTH conversion at 523 K. Besides, only a very weak signal at 184 ppm due to the surface acetyl [10] or the coupling products from acetaldehyde or acetone appeared in the ^13^C CP MAS NMR spectrum, confirming the occurrence of methanol carbonylation. With increasing the reaction temperature to 573 K, more signals appeared at 249, 244, 226, 211, 207, 155, 147,130–134 and 35–10 ppm. According to previous reports, the signals at 207 and 29 ppm were assigned to the formation of acetaldehyde [15], and the signal at 226 ppm was assigned to acetone [33,34]. These results clearly indicated that acetone and acetaldehyde could be formed during the initial process of MTH conversion over H-ZSM-5 catalyst, in line with the methanol-TPSR profiles (Fig. 1a). In addition, the signals at 249, 244, 155 and 147 ppm were due to methylated cyclopentenyl cations [35–41], and the signals at 130-134 ppm were due to aromatics [32]. With the further increase of reaction temperature to 623 K, the amounts of cyclopentenyl cations and aromatics, reflected as the intensities of the signals at *δ*_13C_ at 244-249 and 130-134 ppm, respectively, increased distinctly, while those of acetaldehyde decreased simultaneously. These results indicated that MTH conversion had already proceeded according to the dual-cycle mechanism.

**Fig. 4:**
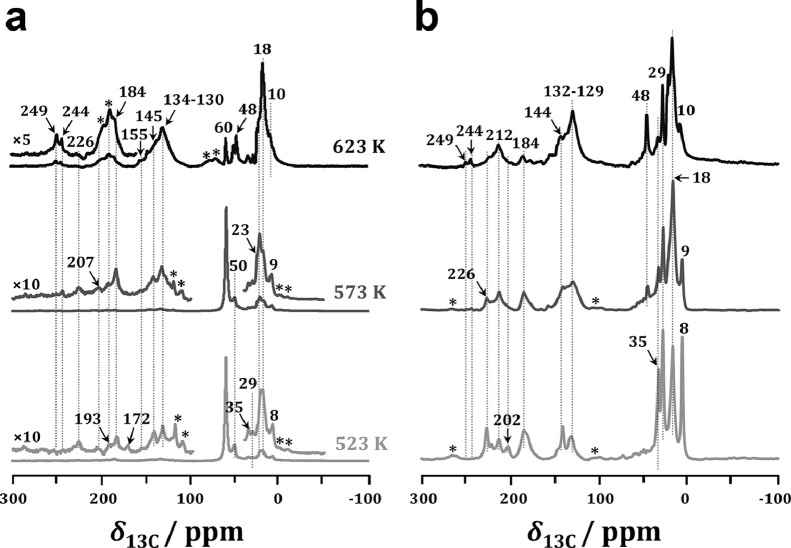
^13^C CP MAS NMR spectra of spent H-ZSM-5 catalysts after MTH conversion with (a) ^13^C-enriched acetaldehyde co-feeding (100 ppm) and (b) ^13^C-enriched acetaldehyde conversion for TOS of 5 min. *: spinning sidebands.

With ^13^C-enriched acetaldehyde co-feeding, the signals at 172, 184 and 193 ppm due to the coupling products derived from acetaldehyde increased distinctly, hinting that acetaldehyde could be rapidly involved in the subsequent aldol condensations (Fig. 4a). Typically, the signal at 172 associated with the signals at 207, 155, 145, 35 and 8 ppm could be due to methylcyclopentenone species [42]. In addition, noticeable amounts of aromatics (*δ*_13C_ at 130–134 ppm) appeared even at a low reaction temperature of 523 K, indicating that acetaldehyde co-feeding could significantly promote the formation of aromatics, and therefore shorten the induction period of MTH reaction, in line with the catalytic results (Fig. 2a). Acetone (*δ*_13C_ =226 ppm) could also be observed with acetaldehyde co-feeding, revealing that acetaldehyde could be converted into acetone *via* the aldol-condensation pathway, in line with the on-line MS monitoring of acetaldehyde conversion (Fig. S2). This was also well supported by ^13^C CP MAS NMR results of acetaldehyde conversion, where an obvious signal at 226 ppm appeared due to the formation of acetone (Fig. 4b).

With increasing reaction temperature, DME and methanol were gradually consumed. Simultaneously, the amounts of the coupling products reflected as the intensities of the signals at *δ*_13C_ =172–212 ppm declined significantly, while those of cyclopentenyl cations and aromatics (*δ*_13C_ at 244–249 and 130–134 ppm) increased. These findings indicated that acetaldehyde could be gradually transferred into cyclopentenyl cations and aromatics *via* the unsaturated aldehyds/ketones as the intermediates. This was supported by *in situ* TP-UV-vis studies of acetaldehyde conversion (Fig. S4), as acetaldehyde (216–220 nm) could be gradually transferred to dienes (235–259 nm), polymethylbenzene (290 and 350–370 nm) and subsequently to polycyclic aromatics (418–425 nm) with increasing reaction temperature.

**Fig. 5:**
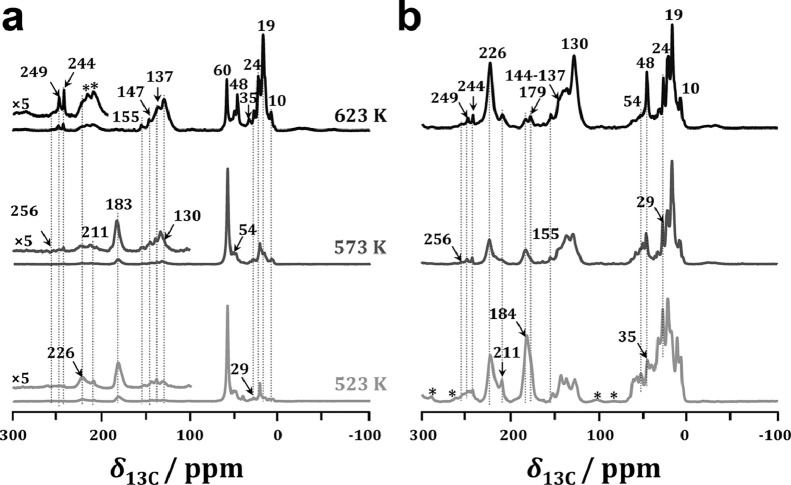
^13^C CP MAS NMR spectra of spent H-ZSM-5 catalysts after MTH conversion with (a) ^13^C-enriched acetone co-feeding (100 ppm) and (b) ^13^C-enriched acetone conversion for TOS of 5 min. *: spinning sidebands.

The aforementioned spectroscopic results clearly indicated that acetic acid as the first C-C containing intermediate could be easily converted to acetone over H-ZSM-5 zeolite (Fig. S2). Therefore, the role of acetone in the MTH conversion should be roughly equivalent to that of acetic acid. In addition, acetaldehyde could be converted to acetone *via* the aldol-condensation pathway (Fig. 4b). Therefore, with the occurrence of acetone, the self and/or cross condensations between acetaldehyde and acetone might take place at the LAS of H-ZSM-5 zeolite. To identify the role of acetone in a deeper manner, ^13^C CP MAS NMR spectroscopy was also employed for MTH conversion with acetone co-feeding. As shown in Fig. 5a, the coupling products of acetone (*δ*_13C_ =183 and 211 ppm) (Table 1) were rapidly formed even at the low reaction temperature of 523 K with acetone co-feeding. Typically, the signals at 211 and 183 ppm could be due to diacetone alcohol and protonated diacetone alcohol, respectively [34,43,44]. In addition, methylated cyclopentenyl cations (*δ*_13C_ at 256, 249, 244, 155 and 147 ppm) and aromatics (*δ*_13C_ at 130–137 ppm) derived from the coupling products appeared, revealing that the MTH conversion had already proceeded according to the dual-cycle mechanism, in line with the catalytic results (Fig. 2c). With the further increasing temperature to 623 K, the amounts of the coupling products reflected as the intensities of the signals at *δ*_13C_=183–211 declined sharply, while those of cyclopentenyl cations (*δ*_13C_ =256–244 ppm) and aromatics (*δ*_13C_ =137–130 ppm) increased synchronously. It clearly indicated that the coupling products could be further converted into dienes and/or aromatics *via* hydrogen transfer and cyclization routes or direct cyclization and dehydration routes [14,36]. This was well supported by ^13^C CP MAS NMR results of ^13^C-enriched acetone conversion (Fig. 5b), where the cyclopentenyl cations and aromatics were rapidly formed even at the low reaction temperature of 523 K, and the amounts of which increased significantly with increasing reaction temperature.

**Table 1:** **Schemes and experimental ^13^C NMR shifts of neutral hydrocarbons and carbenium ions discussed in the present study**.

**Organic species**	**δ_13C_ / ppm**	**Ref.**	**Organic species**	**δ_13C_ / ppm**	**Ref.**
	207 (2)29 (2)	[15]		14.7 (1,2,3)9.8 (4,5,6)	[32]
	224 (2)30 (1)	[33,34]		249.4 (1,3)146.9 (2)48 (4,5)	[[35], [36], [37], [38], [39], [40]]
	29 (1)54 (2)211(3)	[42]		243.2 (1,3)155.7 (2)48 (4,5)	[36,38,40]
	182-188	[34,43,44]		247 (4)143 (3)256 (2)48 (4,5)	[38,40]
	209 (1)142 (2)157 (3)35 (5)	[42]		245 (1,3)150 (2)48 (4,5)	[41]
	209 (1)136 (2)173 (3)8 (6)	[42]		15556244-253	[33]

^1^H MAS NMR measurements were further employed to determine the effects of acetaldehyde and acetone co-feeding on the nature of organic intermediates during the MTH conversion. As shown in Fig. S5, a broad ^1^H MAS NMR signal at approximately 3.5–2.0 ppm due to methoxy species or the adsorbed methanol/DME appeared as the dominant signal at 523 K. With acetaldehyde or acetone co-feeding, two new weak signals at 1.5 and 0.7 ppm due to the methyl signals of the coupling products or polymethylcyclopropane appeared [32], indicating the occurrence of aldol condensation of acetaldehyde or acetone. With increasing reaction temperature to 573 K, new weak signals at about 9.0–7.0 ppm attributed to aromatics started to appear with acetaldehyde or acetone co-feeding. With further increasing reaction temperature to 573 K, the amounts of aromatics, reflected as the intensities of the signals at *δ*_1H_ between 9.0–7.0 ppm, increased sharply, and those of alkenes (*δ*_1H_ at 3.5–2.0) also increased significantly. These findings indicated that acetaldehyde or acetone could effectively promote the formation of alkenes and aromatics, thus inducing the MTH conversion according to the dual-cycle mechanism, in good agreement with the ^13^C CP MAS NMR results (Figs. 4a and 5a).

**Fig. 6:**
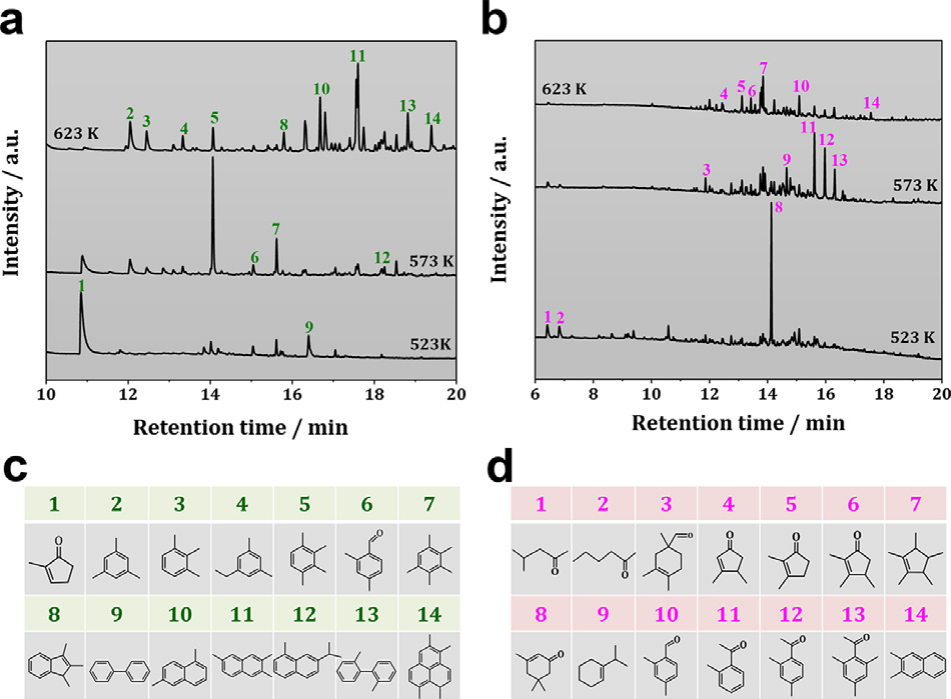
GC-MS chromatograms of organic extracts from H-ZSM-5 catalysts after MTH conversion with (a) acetaldehyde (100 ppm) or (b) acetone (100 ppm) co-feeding for TOS of 5 min. The corresponding structures (**c** and **d**) of the organic compounds occluded in spent H-ZSM-5 catalysts after MTH conversion.

The aforementioned spectroscopic results could also be well supported by GC-MS results. As shown in Fig. 6a, with acetaldehyde co-feeding, 2-MCP was observed as the dominant organic species occluded in H-ZSM-5 catalyst after MTH conversion at 523 K and TOS of 5 min. Meanwhile, a small quantity of methylbenzenes could be observed. With increasing reaction temperature to 573 K, the amount of 2-MCP declined sharply, while polymethylbenzenes emerged as the dominant organic species, indicating that the unsaturated aldehydes/ketones could be gradually transferred to polymethylbenzenes at increasing temperature. With further increasing temperature to 623 K, nearly no 2-MCP could be detected, while naphthalene and polycyclic aromatics appeared as the dominant organic deposits even with a short TOS of 5 min. These results clearly indicated that acetaldehyde co-feeding could significantly promote the aromatics formation (Fig. S6), thus shortening the induction period of MTH conversion, but could also accelerate the accumulation of polycyclic aromatics, causing a rapid catalyst deactivation. It was well supported the catalytic results (Fig. S7), where a rapid catalyst deactivation occurred at 623 K with acetaldehyde co-feeding. For acetone co-feeding, a quite similar variation trend of the unsaturated aldehydes/ketones and aromatics as that of acetaldehyde co-feeding could be observed (Fig. 6b). These findings indicated that the aldol-condensation would be rapidly involved with acetaldehyde or acetone co-feeding, and the formed coupling products could be further transferred into aromatics and induce the MTH conversion according to the aromatic-cycle, in line with previous reports [27,37,45,46].

### 3.3 Roadmap and catalytic cycles of MTH conversion

**Fig. 7:**
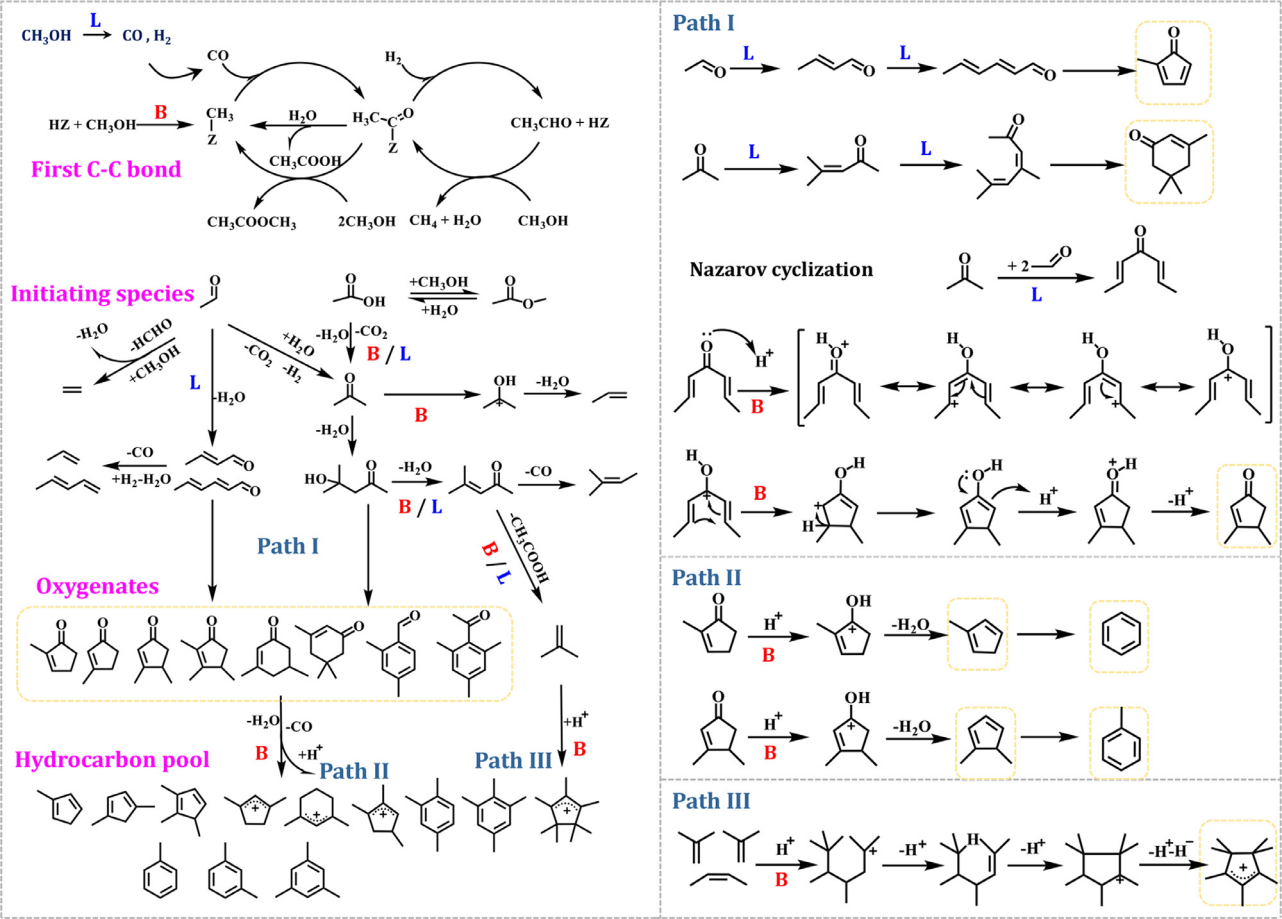
**Proposed roadmap of the MTH conversion over H-ZSM-5 (denoted as HZ) with the participation of both Brønsted (denoted as B) and Lewis acid sites (denoted as L)**.

**Fig. 8:**
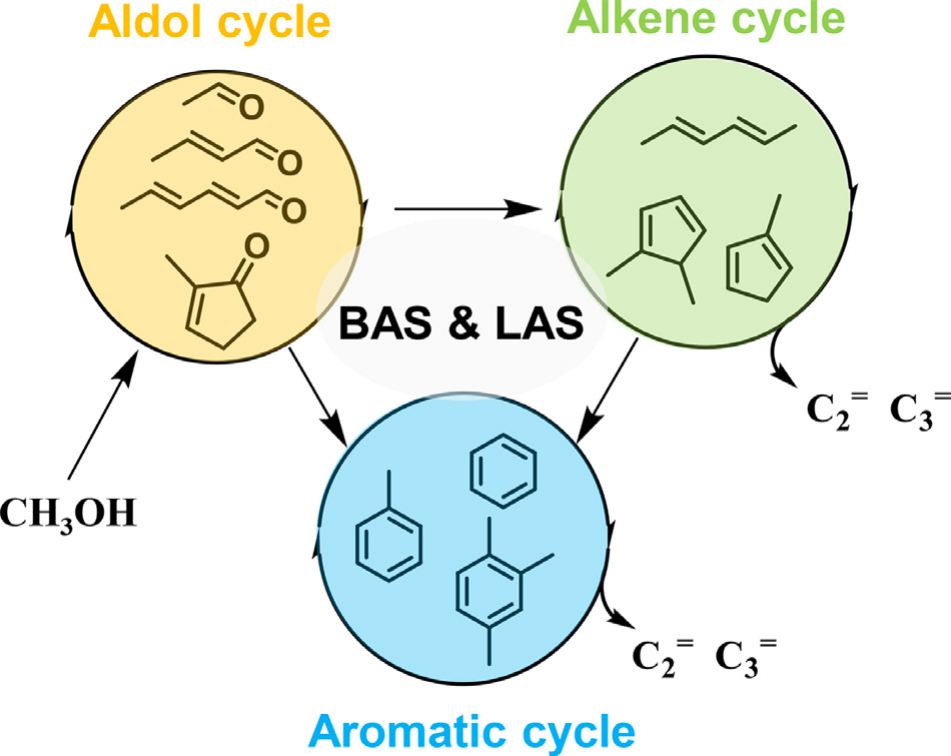
**Simplified three catalytic cycles in MTH conversion over acidic H-ZSM-5 zeolite**.

Based on the aforementioned spectroscopic results, a roadmap of the MTH conversion over H-ZSM-5 catalyst with the participation of both BAS and LAS is proposed in Fig. 7. Firstly, CO and H_2_ could be formed through methanol/formaldehyde dehydrogenation at the LAS (most probably EFAL species), and SMS was formed at the BAS (Si-OH-Al) of H-ZSM-5 zeolite. Subsequently, the surface-bound acetyl species associated with the first C-C bond were formed *via* the carbonylation of SMS and CO over BAS, which could be further converted to acetic acid or methyl acetate in the presence of water and methanol/DME. Additionally, the surface-bound acetyl species could be hydrogenated to acetaldehyde in the presence of H_2_, and the details were available in our previous work [14]. The formation of the aforementioned intermediates containing the first C-C bond, namely acetaldehyde, acetic acid or methyl acetate, could be identified by methanol-TPSR profiles (Fig. 1). In comparison with methyl acetate with much more chemical inertness at low reaction temperature of 523 K, the formed acetaldehyde and acetic acid were immediately involved in the subsequent reactions. The acetic acid could be immediately converted to acetone at BAS/LAS (Fig. 1b). On the other hand, the formed acetaldehyde could be converted to acetone at LAS *via* the aldol-condensation pathway (Figs. S2 and 4). With the involvement of both BAS and LAS, the self and cross condensations of acetaldehyde and acetone would take place, resulting in the formation of chain or cyclic unsaturated aldehydes/ketones (Path 1) (Figs. 3–5, 7) [26]. Subsequently, the unsaturated aldehydes/ketones could be further converted to alkenes *via* the decarbonylation route or cracking route, thus initiating the MTH conversion *via* the alkene-cycle. The formed chain alkenes could be converted to five-/six-ring carbenium ions and aromatics *via* the cyclization, deprotonation, and hydride transfer steps over BAS (Path 3) [32]. In the presence of BAS, the chain coupling products could also be converted to aromatics *via* hydrogen transfer/decarbonylation and cyclization routes or direct cyclization and dehydration routes (Figs. S8–S10) [45]. Meanwhile, the cyclic unsaturated ketones, e.g., 2-MCP and 3,4-diMCP, could be converted to aromatics *via* the protonation, dehydration and isomerization (Path 2), thus initiating the MTH conversion *via* the aromatic-cycle. Finally, with the formation of alkenes and aromatics, dual-cycle mechanism would dominate the MTH conversion. In a word, three cycles, namely aldol-cycle, alkene-cycle and aromatic-cycle, are involved in the roadmap of MTH conversion over H-ZSM-5 zeolite with the participation of both BAS and LAS, and the initial aldol-cycle could bridge the dual-cycle mechanism during the MTH conversion (Fig. 8).

**Fig. 9:**
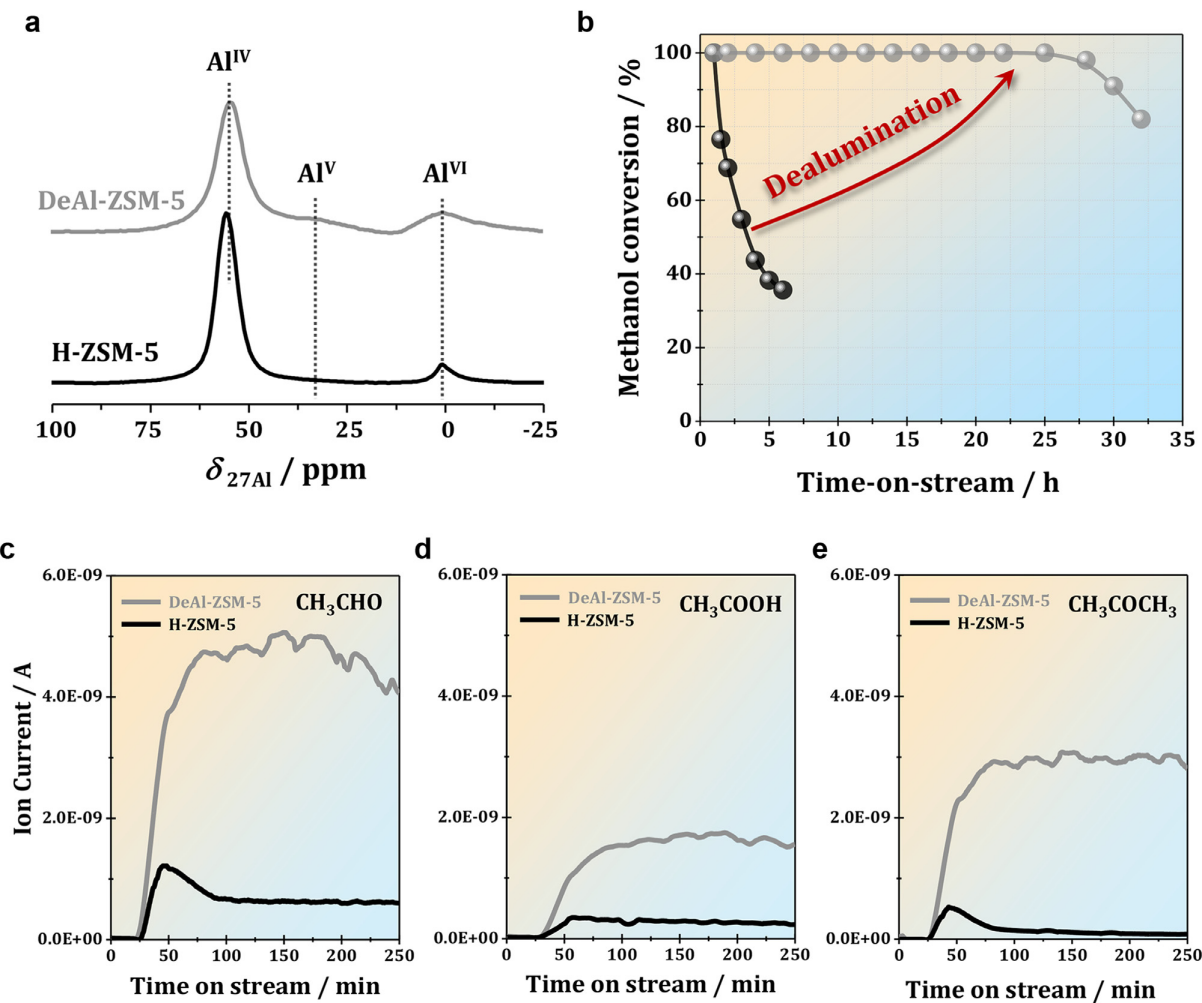
(**a**) ^27^Al MAS NMR spectra of H-ZSM-5 zeolites before and after dealumination (steaming at 673 K for 10 h), and the corresponding MTH activity at 573K (**b**). On-line MS monitoring of acetaldehyde (**c**), acetic acid (**d**) and acetone (**e**) during the MTH conversion over the parent and dealuminated H-ZSM-5 zeolites at 573 K.

To confirm the impacts of LAS (EFAL species) on the organic intermediates and the corresponding catalytic performance in the MTH conversion over H-ZSM-5 zeolite, dealuminated H-ZSM-5 was prepared via high temperature steaming and applied in the MTH conversion. As shown in Fig. 9a, a significant quantity of EFAL (Al^VI^) species (*δ*_27Al_ ≈ 0 ppm) with a noticeable amount of penta-coordinated Al (Al^V^) atoms (*δ*_27Al_ ≈ 32 ppm) were formed by dealumination. Interestingly, in comparison with the parent H-ZSM-5, unprecedented stable MTH activity could be achieved with dealuminated H-ZSM-5 zeolite at the low reaction temperature of 573 K (Fig. 9b), revealing the essential roles of Lewis acid sites in the roadmap of MTH conversion (Fig. 7). The on-line MS analyses clearly demonstrated that much more amounts of oxygen containing unsaturated species, e.g., acetaldehyde, acetic acid and acetone, were formed over dealuminated H-ZSM-5 zeolite (Fig. 9c–e). According to the aforementioned mechanism elaboration, it can be safely concluded that more unsaturated oxygenates were formed over the dealuminated H-ZSM-5 zeolite with a higher amount of LAS (EFAL species) at the very low reaction temperature of 573 K, which could be rapidly converted to HCP species and thus initiated the MTH conversion. That is, promising zeolite catalysts for low temperature MTH reaction could be constructed by tuning the BAS and LAS for future applications.

## 4 Conclusion

In summary, the roadmap of the MTH conversion over H-ZSM-5 zeolite with the participation of BAS and LAS has been clarified *via* a series of spectroscopic approaches. Firstly, the first C-C containing intermediates, namely acetaldehyde, acetic acid and methyl acetate, are identified *via* methanol-TPSR. Acetone, as an intermediate of acetic acid and acetaldehyde, is disclosed *via* acetic acid TPSR and ^13^C MAS NMR spectroscopy. Subsequently, three catalytic cycles, namely aldol-cycle, alkene-cycle and aromatic-cycle, are confirmed to be involved in the MTH conversion with the participation of both BAS and LAS. On the LAS, the self and cross condensations of acetaldehyde and acetone take place, leading to the formation of chain or cyclic unsaturated aldehydes/ketones. According to the cracking route, the unsaturated ketones can be converted to alkenes and release acetic acid/acetaldehyde again, thus inducing the first aldol-cycle. On the other side, the unsaturated aldehydes/ketones can be further converted to alkenes *via* the decarbonylation route, triggering the MTH conversion via the alkene-cycle over the BAS. Meanwhile, the coupling products can be converted to aromatics *via* hydrogen transfer and cyclization routes or direct cyclization and dehydration routes, initiating the MTH conversion *via* the aromatic-cycle. The full knowledge on the progressive steps and catalytic cycles in MTH reaction is extremely important for the future catalyst design. Promising zeolite catalysts for low temperature MTH reaction can be constructed by tuning the BAS and LAS for future applications.

## Declaration of Competing Interest

The authors declare that they have no conflicts of interest in this work.
