# Supplementary material for: Progressive steps and catalytic cycles in methanol-to-hydrocarbons reaction over acidic zeolites
Source: Fundam Res. 2021 Aug 16;2(2):184–92. doi: 10.1016/j.fmre.2021.08.002 (PMC11197791; doi:10.1016/j.fmre.2021.08.002)
Supplement: Supplementary file 1 [file mmc1.docx]

**Supporting Information**

**Progressive steps and catalytic cycles in methanol-to-hydrocarbons reaction over acidic zeolites**

Liu Yang^1^, Chang Wang^1^, Weili Dai^1^*, Guangjun Wu^1^, Naijia Guan^1,2^, Landong Li^1,2^*

^1^ School of Materials Science and Engineering, Nankai University, Tianjin 300350, P.R. China

^2^ Frontiers Science Center for New Organic Matter & Key Laboratory of Advanced Energy Materials Chemistry of Ministry of Education, College of Chemistry, Nankai University, Tianjin 300071, P.R. China

**Corresponding Authors**

* E-mail: weilidai@nankai.edu.cn (W. Dai); [lild@nankai.edu.cn](mailto:lild@nankai.edu.cn) (L. Li)

**Fig. S1.** ^13^C CP MAS NMR spectrum of ^13^C-2-acetone adsorption on dehydrated H-ZSM-5 zeolite followed by room temperature evacuation. Asterisks denote spinning sidebands.

^13^C-2-acetone (CH_3_^13^COCH_3_) was utilized as a probe molecule in ^13^C CP MAS NMR spectroscopy to distinguish the Brønsted acid sites of different strengths (δ_13C_ = 210-225 ppm) and the Lewis acid sites (δ_13C_ = 225-266 ppm) in zeolite sample. **Figure S1** presents the spectrum of ^13^C-2-acetone adsorption on dehydrated H-ZSM-5 zeolite followed by room temperature evacuation. The signal of δ_13C_ = 209 ppm is due to ^13^C-2-acetone adsorption at SiOH groups. The signal of δ_13C_ = 225 ppm is characteristic for acetone adsorption at the strong Brønsted acid sites, i.e., Si(OH)Al, while the low-field shoulder at δ_13C_= 234 ppm corresponds to acetone adsorption at weak Lewis acid sites. That is, both Brønsted and Lewis acid sites exist in the H-ZSM-5 zeolite employed.

**Fig. S2.** On-line MS monitoring of the selected reaction products during acetaldehyde conversion over H-ZSM-5 at 523K.

**Fig. S3.** *In situ* UV-vis spectra of acetic acid (**a**) and acetone (**b**) conversion over H-ZSM-5 catalyst at 523 K with the TOS of 30 min.

**Fig. S4**. Temperature-programmed UV-vis spectra obtained during acetaldehyde conversion over H-ZSM-5 zeolite at reaction temperatures of 293-673K.

**Fig. S5.** ^1^H MAS NMR spectra of spent H-ZSM-5 catalysts after MTH conversion without and with acetaldehyde or acetone co-feeding (100 ppm) for TOS of 5 min.

**Fig. S6.** (**a**) GC-MS chromatograms of organic extracts from H-ZSM-5 catalysts after MTH conversion before and after acetaldehyde (75 ppm) co-feeding for TOS of 30 min. The corresponding structures (**b**) of the organic compounds occluded in spent H-ZSM-5 catalysts after MTH conversion.

**Fig. S7.** Methanol conversion over H-ZSM-5 zeolite without and with acetaldehyde co-feeding (100 ppm) at 623 K.

**Fig. S8.** Proposed route for acetone conversion to aromatics and the initiation of MTH conversion via aromatic-cycle (**a**). On-line MS monitoring of the selected reaction products during acetone conversion over H-ZSM-5 at 523 K (**b** and **c**).

**Fig. S9.** Proposed route for the cross condensations between acetaldehyde and acetone to aromatics.

**Fig. S10.** Proposed route for the cross condensations between acetaldehyde and acetone to aromatics.

| **Table S1**. The m/z values for chemicals employed in this study and their relative intensities | |
| --- | --- |
| H_2_ | 2 (100%), 1(2%) |
| He | 4 (100%) |
| CO | 28 (100%), 12(6%), 16(2%) |
| ^13^CO | 29(100%), 13(6%), 16(2%) |
| CH_4_ | 16(100%), 15(90%), 14(20%) |
| C_2_H_4_ | 28(100%), 27(64%), 26(55%), 25(9%) |
| ^13^C_2_H_4_ | 30(100%), 29(64%), 28(55%), 27(9%) |
| CH_3_OH | 31 (100%), 32 (75%), 29(45%), 15(14%), 30(8%) |
| ^13^CH_3_OH | 32 (100%), 33 (75%), 30 (45%), 16(14%), 31(8%) |
| C_3_H_6_ | 41 (100%), 39(74%), 42 (71%), 27(39%) |
| ^13^C_3_H_6_ | 44 (100%), 42(74%), 45 (71%), 29(39%) |
| CH_3_CHO | 29(100%), 44(85%), 43(50%) |
| ^13^CH_3_^13^CHO | 30(100%), 46(85%), 45(50%) |
| CH_3_COCH_3_ | 43(100%), 58(23%), 15(12%) |
| ^13^CH_3_^13^CO^13^CH_3_ | 45(100%), 61(23%), 16(12%) |
| CH_3_-O-CH_3_ | 45 (100%), 46 (61%), 29(39%) |
| ^13^CH_3_-O-^13^CH_3_ | 47 (100%), 48 (61%), 30 (39%) |
| CH_3_COOH | 43 (100%), 45(91%), 60(75%) |
| ^13^CH_3_^13^COOH | 45 (100%), 46(91%), 62(75%) |
| CH_3_COOCH_3_ | 43 (100%), 74(26%), 42(15%) |
| ^13^CH_3_^13^COO^13^CH_3_ | 45 (100%), 77(26%), 44(15%) |
| 2-MCP | 67 (100%), 96(80%), 53(48%) |
| 2-MCP(^13^C) | 72 (100%), 102(80%), 57(48%) |
| PentaMBs | 133(100%), 148(55%), 134(12%) |
| PentaMBs(^13^C) | 144(100%), 159(55%), 145(12%) |
| The m/z values of the species employed in this work were marked in **red** | |
